# Co-creating green steps: APIM evidence of mutual influence on pro-environmental behavior in travel pairs

**DOI:** 10.3389/fpsyg.2026.1730412

**Published:** 2026-03-02

**Authors:** Bopeng Yu, Jing Shi

**Affiliations:** School of Educational Science, Shenyang Normal University, Shenyang, China

**Keywords:** actor-partner interdependence, comparative research, environmental values, pro-environmental behavior, pro-environmental identity

## Abstract

This study investigated the dyadic mechanisms underlying pro-environmental behavior in travel partnerships, focusing on differences between planner-dominated and co-planner dyads. Using data from 350 travel dyads, we applied the actor-partner interdependence model (APIM) and the actor-partner interdependence mediation model (APIMeM) to analyze mutual influence patterns. In planner-dominated dyads, results revealed that an individual's environmental values significantly predicted their own pro-environmental behavior, while the planner's environmental values also significantly predicted the follower's behavior. Conversely, in co-planner dyads, individuals' environmental values predicted both partners' pro-environmental behavior. Pro-environmental identity emerged as a key mediator, bridging the relationship between environmental values and behavior across plan-making divisions. Specifically, in planner-dominated dyads, the planner's pro-environmental identity mediated the link between their environmental values and both their own and the follower's behavior. In co-planner dyads, each partner's pro-environmental identity independently mediated their environmental values and behavior. These findings highlight that power dynamics critically shape the efficacy of pro-environmental influence in travel pairs. The study underscores a fundamental shift from hierarchical compliance in planner-dominated dyads to active co-creation of green behaviors in equal partnerships, driven by mutual influence and shared identity. Consequently, promoting collaborative decision-making emerges as a pivotal strategy for embedding sustainability into the core of travel experiences.

## Introduction

In the urgent calculus of planetary survival, our pro-environmental behavior is no longer an altruistic choice, it is a survival stake where every individual action compounds into collective destiny. Pro-environmental behavior is the behavior of individuals that contribute toward environmental preservation (Turaga et al., 2010). This kind of behavior covers multiple fields, including but not limited to resource conservation, pollution reduction, biodiversity protection, and support for environmental protection policies (Carrico, 2022; Dewi and Dian, 2018; Sawitri et al., 2015). With the popularity of group travel in modern times, comes its negative environmental impact. Therefore, enhancing tourist pro-environmental behavior, which refers to pro-environmental actions demonstrated during travel, is of particular importance. However, there is still much to be explored regarding how to enhance their pro-environmental behavior.

Previous researches mainly focused on individual-level factors and social aspects of pro-environmental behavior (Smith et al., 2021; Zeng et al., 2023), but it neglected the dual dynamics that occur in interpersonal relationships. The environmental cognition theory suggests that individuals' perceptions and attitudes toward the environment are influenced by their peers, and together they shape each other's actions regarding environmental protection (Gao et al., 2019), especially in travel partnerships (Manner-Baldeon et al., 2023). Individuals tend to enhance their own pro-environmental behaviors, such as waste sorting, low-carbon travel, and pro-environmental donations, mainly based on social norms and the pro-environmental behaviors exhibited by those around them (Zhang and Yan, 2025; Zhu and Wang, 2021). Traditional individual-centered models fail to capture these interdependent dynamics, whereas the Actor-Partner Interdependence Model (APIM) adequately offer an appropriate statistical framework to overcome this limitation. APIM allows for the distinction between actor effects (the influence of an individual on their own outcomes) and partner effects (the influence of an individual on their partner's outcomes), making it ideally suited for dyadic data analysis. Building on APIM, APIMeM further enables the examination of mediation effects within dyads, which is crucial for understanding the psychological mechanisms underlying interpersonal influence (Cook and Kenny, 2005). Therefore, this study employs APIM and APIMeM to achieve three primary objectives. (1) To examine the actor and partner effects of environmental values on pro-environmental behaviors within travel dyads. (2) To investigate the mediating role of pro-environmental identity in the relationship between environmental values and pro-environmental behavior. (3) To compare the different pathways between planner-dominated and co-planner dyads, thereby elucidating how power structures facilitate or inhibit pro-environmental behavior. Investigating the mechanisms of pro-environmental behavior in travel partnerships from a dyadic perspective has theoretical and practical significance for environmental protection and ecological balance.

## Literature review

### Environmental values

Considering environmental protection and pro-environmental behavior, discussion of the matter of environmental values which refer to the beliefs and attitudes that people hold toward the environment is inevitable (Yuksel, 2021). Environmental values influence how people interact with the environment (Scoville, 1995; Tumanggor et al., 2023). It encompasses anthropocentrism, biocentrism, ecocentrism and holism. In the binary context of travel companions, the practice of environmental values is reflected in the formulation of travel plans. Interdependence theory elucidates the intricate mutual impacts inherent in dyadic relationships (Kelley et al., 2003). In this view, an individual's values can influence others actions. For instance, individuals with strong environmental values may interpret others' pro-environmental behavior as social norms, thereby strengthening their own sense of moral obligation and enhancing their engagement in pro-environmental actions (Capiene et al., 2022). The mutual influence of values is a dynamic process, but few studies have focused on its dynamics from an empirical perspective. Especially the travel setting, characterized by frequent and close social interaction, creates an environment where individuals' values interact and subsequently guide their mutual pro-environmental behavior.

### Pro-environmental identity mediates the relationship between both parties' environmental values and pro-environmental behavior

The Theory of Planned Behavior states that an individual's identity recognition influenced by their own values and social norms prompts the individual to exhibit certain behaviors (Ajzen, 1985; Ramos, 2024). Pro-environmental identity refers to an individual's self-definition as a member of the pro-environmental group (Turner and Reynolds, 2003). It serves as an important link between environmental values and pro-environmental behavior. According to the social identity theory (Ozyilmaz and Koc, 2022), individuals with a strong environmental value system are more likely to consider themselves as environmentalists and possess a positive environmental identity (Barbarossa et al., 2017), which leads to pro-environmental behavior (Shekhovtsova, 2020). According to the interdependence theory (Kelley et al., 2003), an individual's environmental values may influence partners' pro-environmental identity and pro-environmental behavior. A person who values environmental protection will make those around them more environmentally conscious (Merin Adelin and Dimas Angga, 2024), and this will also foster the formation of a pro-environmental identity (Zhao et al., 2023). Therefore, we considered that pro-environmental identity plays a mediating role between their own and others' environmental values and pro-environmental behavior.

### Different power structures lead to path differences between the planner-dominated dyads and co-planner dyads

When applying the actor-partner interdependence model (APIM) to the study of tourism behavior, it remains critical to incorporate the role positioning of travel companions (specifically, power distribution). In traditional APIM frameworks, interacting dyads have typically been conceptualized within contexts of equal power hierarchy (e.g., parent-child, marital, or romantic partnerships). However, tourism contexts encompass dual scenarios those characterized by equal power dynamics (e.g., joint formulation of travel plans) and those marked by unequal power relations (e.g., between plan-makers and followers). Comparing these two distinct APIM patterns not only holds significant implications for research on tourist psychology and pro-environmental behavior but also enriches understanding of the model's application scenarios and the interpretation of its outcomes. Under conditions of unequal power, follower behavior is more likely to emerge through external regulation (Magee, 2020; Schwab and Singh, 2024). Conversely, in contexts of equal power, mutual cognitive internalization occurs, leading to the formation of persistent behavioral patterns (Núñez-Regueiro, 2024).

Tourism planning is not merely about determining the destination and activities. It also involves contributing to the Sustainable Development Goals (Khizar et al., 2023). Therefore, when formulating a travel plan, it is necessary to take into account the implementation of pro-environmental behavior. There are advantages and disadvantages to both making travel plans independently and collaborating to make them. Making travel plans independently is more convenient and represents the planner's environmental values, which in turn influences pro-environmental behavior. The process of jointly formulating a travel plan requires in-depth cooperation from both parties. In such a social interaction scenario, it can enhance pro-environmental behavior (Zheng et al., 2019; Zhu et al., 2022). At the same time, in the context of jointly completing the plan, the values of both parties are more likely to be exposed, which leads to conflicts in values and subsequently affects each other's sense of identity (Kühler, 2021). Therefore, compared to dictatorship, through cooperative planning of travel it may lead to a special path. Under the cooperative condition, the environmental values of both parties can have a greater impact on their pro-environmental behavior and pro-environmental identity. Meanwhile, dictatorial and cooperative decision-making reflect different power structures, and they have a significant impact on decision-making. Decision-makers tend to make decisions that are favorable to themselves, and the resulting pro-environmental behavior will overly rely on the values of the decision-makers themselves (Li et al., 2024). On the contrary, in cooperative decision-making scenarios, through social interaction, people are often able to enhance their eco-friendly travel decisions (Zheng et al., 2019).

Consequently, we utilized the Actor-Partner Interdependence Model (APIM; Cook and Kenny, 2005) and the Actor-Partner Interdependence Mediation Model (APIMeM; Ledermann et al., 2011) to systematically analyze both intra-individual (actor effects) and inter-individual (partner effects) pathways, and conducted path difference analysis for the two different tourism planning approaches of dictatorship and cooperation.

## Method

### Participants

We utilized APIM POWER (Ackerman and Kenny, 2016; effect size *f*^2^ = 0.25, power = 0.80, α = 0.05) to determine the minimum sample size of 121 dyads. We recruited 375 pairs of people through online social media platforms, who had traveled together before. After removing 25 invalid dyads (defined as those with insufficient response time or failure to pass attention-check items), data from 350 dyads were analyzed formally. The sample comprised 64.6% female and 35.6% male participants, with the majority aged 18 to 35 years. In terms of travel style preferences, 67.7% of participants reported that they preferred traveling with companions, while 32.3% indicated a preference for solo travel. Participants were assigned to one of two mutually exclusive groups based on their responses to a key travel decision-making item: “In your most recent trip together, who was primarily responsible for making key decisions (e.g., destination selection, itinerary planning, accommodation booking)?” Responses were recorded on a 5-point scale (1 = I made all the decisions, 2 = I made most of the decisions, 3 = We made decisions equally, 4 = My partner made most of the decision, 5 = My partner made all the decisions). Based on this measure, Planner-Dominated Dyads were defined as those in which one partner selected option 1 or 2 and the other partner selected option 4 or 5. Co-Planner Dyads were defined as those in which both partners selected option 3. Followed this classification procedure, 159 dyads were identified as Planner-Dominated Dyads and 191 dyads as Co-Planner Dyads.

### Measures

#### Environmental values

Environmental values were assessed using the New Environmental Paradigm Scale Revised (Dunlap et al., 2000), comprising 15 items (e.g., “Humans are severely abusing the environment”) rated on a 5-point scale (1 = strongly disagree to 5 = strongly agree).

#### Pro-environmental identity

Pro-environmental identity was measured via the revised version of the Pro-environmental Identity Questionnaire (Bamberg et al., 2015; Wallis and Loy, 2021), comprising 3 items (e.g., “I identify with others who engage in environmental protection”) rated on a 5-point scale.

#### Pro-environmental behavior.

Pro-environmental behavior was evaluated using the revised version of Environmental Responsible Behavior Questionnaire (Chiu et al., 2013), comprising 7 items (e.g., “I sort my trash at the travel site”) rated on a 7-point scale.

#### Methods of analysis

APIM is a statistical framework to analyze dyadic data where individuals are interdependent, such as couples, parent-child pairs, or colleagues. APIM distinguishes between actor effects (an individual's predictors influence their own outcomes) and partner effects (an individual's predictors influence their partner's outcomes). This model addresses the non-independence of dyadic data, which violates traditional statistical assumptions, and is widely applied in psychology, sociology, and healthcare research to study relational dynamics. Building on APIM, APIMeM integrates mediation pathways to explore how actor/partner effects operate through intervening variables.

SPSS 26.0 was utilized to conduct descriptive statistics and correlation analysis. AMOS 26.0 was utilized for the Actor-Partner Interdependence Model. The travel partner was the unit of analysis, and the models were estimated using the maximum likelihood (ML) estimator. The actor and partner effects were tested using bias-corrected bootstrapped 95% confidence intervals with 5,000 resamples.

## Results

Descriptive statistics and correlations are shown in Table 1. Cronbach' s α are shown in parentheses. All the measures had adequate internal consistency and significantly correlated with each other. The data were fit for further exploration of path analysis.

**Table 1:** Means standard deviations, and correlations.

**Variables**	***M***	***SD***	**1**	**2**	**3**
1 Environmental values	3.30	0.91	(0.93)		
2 Pro-environmental identity	3.30	1.23	0.33^***^	(0.88)	
3 Pro-environmental behavior	3.33	1.18	0.35^***^	0.40^***^	(0.94)

^***^p<*0.001*.

### Invariance tests and distinguishability comparisons

The invariance tests and distinguishability comparisons were conducted to examine for dyadic models across travel relationships. As shown in Table 2, compared with the baseline model, ΔCFI was all greater than 0.010, and the model invariance tests were not supported (Chen, 2007). Therefore, there were differences in the paths between planner-dominated dyads and co-planner dyads. Meanwhile, the results of the distinguishability test indicated that in APIM and APIMeM, the planner-dominated dyads are distinguishable pairs, while the co-planner dyads are indistinguishable pairs (Ledermann et al., 2011).

**Table 2:** Model comparison.

**Invariance tests and distinguishable tests**	**df**	**CMIN**	***p***	**ΔNFI**	**ΔIFI**	**ΔCFI**	**ΔGFI**
**APIM**							
Structural weights	4	11.546	0.021	0.076	0.077	0.054	0.016
Structural covariances	7	30.610	0.000	0.200	0.203	0.168	0.040
Structural residuals	9	52.327	0.000	0.342	0.347	0.308	0.064
Indistinguishable vs. Distinguishable (Planner-dominated dyads)	2	13.200	0.001	0.183	0.183		
Indistinguishable vs. Distinguishable (Co-planner dyads)	2	2.743	0.254	0.034	0.034		
**APIMeM**							
Structural weight	12	25.970	0.011	0.064	0.064	0.037	0.024
Structural co variances	15	45.034	0.000	0.110	0.110	0.079	0.040
Structural residuals	21	75.611	0.000	0.185	0.185	0.144	0.063
Indistinguishable vs. Distinguishable (Planner-dominated dyads)	6	20.036	0.003	0.124	0.124		
Indistinguishable vs. Distinguishable (Co-planner dyads)	6	8.767	0.187	0.036	0.036		

### APIM

The APIM was conducted to examine the relations between environmental values and pro-environmental behavior in two dyads.

**Figure 1:**
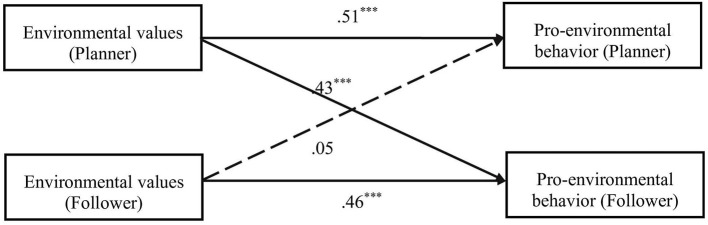
APIM of environmental values and pro-environmental behavior in planner-dominated dyads.

**Figure 2:**
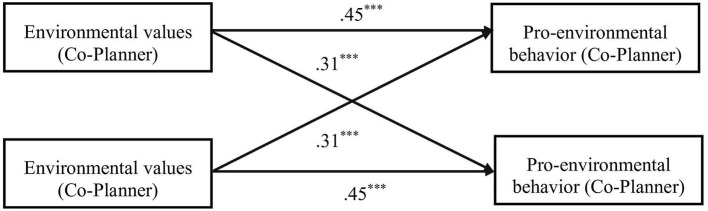
APIM of environmental values and pro-environmental behavior in co-planner dyads.

As shown in Figures 1, 2, actor effects were both significant for planner-dominated dyads. Partner effects were significant for follower but planner, indicating that planners' environmental values directly influence followers' pro-environmental behavior but followers' environmental values did not influence planners' pro-environmental behavior. Actor effects and partner effects were both significant for co-planner dyads.

### APIMeM

**Figure 3:**
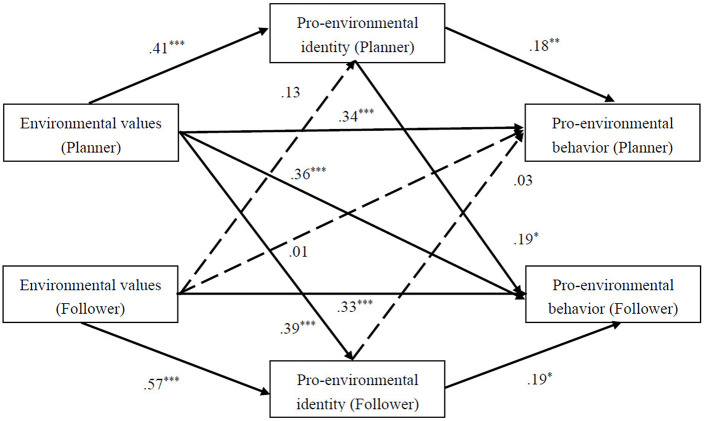
APIMeM of Pro-environmental identity in the Relationship Between Environmental values and Pro-environmental behavior in planner-dominated dyads.

**Figure 4:**
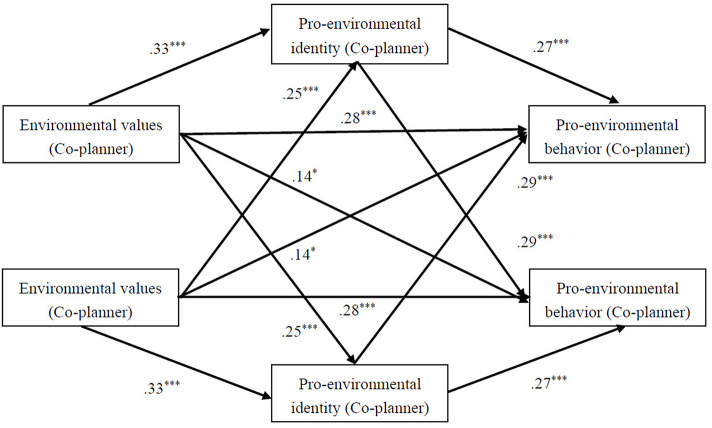
APIMeM of Pro-environmental identity in the Relationship Between Environmental values and Pro-environmental behavior in co-planner dyads.

APIMeM was used to investigate the relations between environmental values, pro-environmental identity and pro-environmental behavior in two dyads (see Figures 3, 4).

Bootstrapping analysis was presented in Tables 3, Table 4. For planner-dominated dyads. Pro-environmental identity mediated the links between individuals' environmental values and their pro-environmental behavior, indicating significant actor mediation effects. Both of their pro-environmental identity mediated the links between planners' environmental values and followers' pro-environmental behavior, indicating significant followers' partner mediation effects. Intriguingly, followers' pro-environmental behavior was concurrently mediated by pro-environmental identity of both, while planners' pro-environmental behavior was mediated only by their own pro-environmental identity and the follower's environmental values did not influence the planner's pro-environmental behavior. Actor mediation effects and partner mediation effects were both significant for co-planner dyads.

**Table 3:** Bootstrapping analysis results for APIMeM in planner-dominated dyads.

**Type of effect**	**Pathway**	**Effect**	**SE**	**95%CI**
Actor effect (Planner)	Total effect	0.43	0.09	[0.22,0.58]
	Direct effect	0.34	0.08	[0.17,0.47]
	Total indirect effect	0.09	0.04	[0.02,0.15]
	Environmental values (Planner) → Pro-environmental identity (Planner) → Pro-environmental behavior (Planner)	0.08	0.03	[0.02,0.16]
	Environmental values (Planner) → Pro-environmental identity (Follower) → Pro-environmental behavior (Planner)	0.01	0.03	[−0.05,0.06]
Partner effect (Planner)	Total effect	0.05	0.07	[−0.10,0.19]
	Direct effect	0.01	0.08	[−0.14,0.19]
	Total indirect effect	0.04	0.04	[−0.05,0.13]
	Environmental values (Follower) → Pro-environmental identity (Planner) → Pro-environmental behavior (Planner)	0.02	0.02	[−0.01,0.09]
	Environmental values (Follower) → Pro-environmental identity (Follower) → Pro-environmental behavior (Planner)	0.02	0.04	[−0.07,0.09]
Actor effect (Follower)	Total effect	0.46	0.10	[0.28,0.65]
	Direct effect	0.33	0.10	[0.12,0.52]
	Total indirect effect	0.13	0.05	[0.05,0.24]
	Environmental values(Follower) → Pro-environmental identity (Follower) → Pro-environmental behavior(Follower)	0.11	0.05	[0.03,0.22]
	Environmental values (Follower) → Pro-environmental identity (Planner) → Pro-environmental behavior (Follower)	0.03	0.02	[−0.004,0.10]
Partner effect (Follower)	Total effect	0.51	0.13	[0.26,0.77]
	Direct effect	0.36	0.13	[0.13,0.61]
	Total indirect effect	0.15	0.06	[0.05,0.29]
	Environmental values (Planner) → Pro-environmental identity (Follower) → Pro-environmental behavior (Follower)	0.07	0.04	[0.02,0.17]
	Environmental values (Planner) → Pro-environmental identity (Planner) → Pro-environmental behavior (Follower)	0.08	0.05	[0.01,0.18]

CI, confidence interval.

**Table 4:** Bootstrapping analysis results for APIMeM in co-planner dyads.

**Type**	**Pathway**	**Effect**	**SE**	**95%CI**
Actor	Total effect	0.44	0.08	[0.31,0.57]
	Direct effect	0.28	0.08	[0.15,0.40]
	Total indirect effect	0.16	0.03	[0.10,0.23]
	Actor-Actor indirect effect	0.09	0.02	[0.04,0.16]
	Partner-Partner indirect effect	0.07	0.02	[0.03,0.13]
Partner	Total effect	0.30	0.09	[0.17,0.43]
	Direct effect	0.14	0.08	[0.01,0.26]
	Total indirect effect	0.16	0.03	[0.11,0.23]
	Actor-Partner indirect effect	0.10	0.02	[0.04,0.16]
	Partner-Actor indirect effect	0.07	0.02	[0.03,0.13]

CI, confidence interval.

### Comparison of coefficients between planner-dominated and co-planner models

As shown in Table 5, comparison of coefficients was used to investigate the differences in path coefficients between Planner-dominated model and Co-planner model (Duncan, 2014). In terms of the partner effect, both the total effect and indirect effect in the co-planner scenario were significantly greater than the planner-dominated scenario.

**Table 5:** Comparison of coefficients between planner-dominated and co-planner models.

**Path comparison type**	**Planner-dominated**		**Co-planner**		***p***
	**Effect**	**SE**	**Effect**	**SE**	
Actor Total effect (Planner vs. Co-planner)	0.43	0.09	0.44	0.08	0.934
Actor Direct effect (Planner vs. Co-planner)	0.34	0.08	0.28	0.08	0.595
Actor Indirect effect (Planner vs. Co-planner)	0.09	0.04	0.16	0.03	0.161
Partner Total effect (Planner vs. Co-planner)	0.05	0.07	0.30	0.09	0.028
Partner Direct effect (Planner vs. Co-planner)	0.01	0.08	0.14	0.08	0.250
Partner Indirect effect (Planner vs. Co-planner)	0.04	0.04	0.16	0.03	0.016

## Discussion

We utilized APIMeM to examine the mechanisms by which environmental values affect pro-environmental behavior in different forms of travel partner. Meanwhile, comparative analysis was conducted to examine the differences in paths that emerge under different forms of plan formulation. The results indicated that individuals' environmental values directly influence their pro-environmental behavior. For those travel plans formulated by planners, the planners' own pro-environmental identity affects both their own and their travel partners' pro-environmental behavior, thereby forming an influence path dominated by the planners. For those travel plans jointly formulated by both parties, the pro-environmental identity of each party influences their own and their travel partners' pro-environmental behavior, thus forming a two-way influencing path. These findings enhance our comprehension of value transmission mechanisms in group travel relationships and provide important insights for cultivating environmentally friendly travel behaviors in an era of widespread tourism.

### Direct driving role of environmental values in pro-environmental behavior

Our findings showed that travel partners' environmental values have a direct actor effect on pro-environmental behavior in different plan scenarios. This result is consistent with previous studies (Balunde and Perlaviciute, 2023; Xu et al., 2022). Environmental values are an important internal factor guiding individual pro-environmental behavior. They influence an individual's attitude toward the environment, environmental cognition, and identity, thereby promoting or inhibiting the occurrence of pro-environmental behavior (Chwialkowska et al., 2020; Karp, 1996). At the same time, environmental values can influence an individual's perception and emotional response to environmental issues, enhancing their environmental awareness and thereby encouraging them to engage in more pro-environmental behavior (Chwialkowska et al., 2020).

### Contextual dependence of partner effects: power structures as drivers of path differences

However, the partner effect demonstrated context-specificity. In planner-dominate dyads, only planners' environmental values could directly induce partners' pro-environmental behavior. In co-planner dyads, environmental values could directly induce both partners' pro-environmental behavior. This disparity reveals that the power structure divide has shaped a completely different picture of environmental behavior transmission. In the dominant pair (planner-dominated dyads), the environmental values of the planner act as the central hub of behavior, and their radiating power simultaneously covers both themselves and their partner's environmental behaviors, forming a single-pole-driven two-body transmission paradigm. This cross-boundary behavioral control corroborates the behavior approach system of social power theory (Brauer and Bourhis, 2006). When decision-making power is highly centralized, the value system of the dominant party can directly cross individual boundaries to reconfigure the behavioral choices of the partner (Galinsky et al., 2003). However, collaborative pairs (co-planned dyads) demonstrate the equality principle of a democratic system. Both parties' environmental values mutually influence their own and each other's pro-environmental behaviors, and through forms such as social interaction, they are enhanced in a dual-track parallel manner (Balunde and Perlaviciute, 2023; Cao et al., 2025). This result indicated that power sharing has removed the shackles on the dissemination of values, enabling them to mutually reinforce each other's behaviors in an equal field, suppled the mechanical assumption of the traditional model that separates actor and partner. This fundamental divergence in pathways elucidates how power structures reconfigure the mechanisms of interpersonal influence. Under power asymmetry, influence is unidirectional and instrumental, hinging on the planner's attributes. But under power balance, it transforms into a bidirectional, internalized process achieved through social interaction and consensus building.

### Context-specific mediating mechanism of pro-environmental identity

The mediating mechanism of pro-environmental identity also demonstrated context-specificity. For planner-dominate dyads, planners' environmental values could directly induce partners' pro-environmental behavior but also indirectly influence it through pro-environmental identity. For co-planner dyads, environmental values did directly and indirectly influence pro-environmental behavior through pro-environmental identity. This outcome indicates that various planning models will produce distinct behavioral results. When travel companions adopt different decision-making patterns, they are essentially activating completely distinct social psychological mechanisms. For planner-dominated dyads, the planner utilizes their decision-making power to exert psychological and behavioral influence on the follower. However, this form of top-down influence often yields effects that are less durable and inherently constrained by the planner's personal commitment and presence. In contrast, within co-planner dyads, the process of joint planning enhances social interaction, which facilitates a deeper internalization of a shared pro-environmental identity between partners (Jaeger and Schultz, 2017). This mutual internalization through identity is the core psychological process that enhances pro-environmental behavior where pro-environmental behavior from a shared sense of environmental self-identity, rather than being imposed or merely adopted. Consequently, the mediating role of pro-environmental identity is not static but is activated and shaped by the power dynamics. Therefore, the pivotal shift from absolute power domination to shared power is key to fostering a shared pro-environmental identity and enhancing pro-environmental behavior.

### Planner-dominated dyads: dual drivers and limitations under power imbalance

In the planner-led combination, the power imbalance enables the planner to simultaneously employ instrumental influence (by controlling resources to directly request behavioral changes) and referential power (by setting an example to elicit the partner's recognition), forming a dual-track drive of “command to internalization” (Anderson and Brion, 2014; Yin et al., 2021). This mode is efficient but fragile that relies heavily on the planner's personal environmental values, which directly and promptly influence the follower's pro-environmental identity and behavior, thereby achieving a significant short-term boost in the follower's pro-environmental actions. However, the partner's behavior is mostly driven by compliance rather than genuine acceptance, and the resulting behavioral reproducibility is relatively low (Kim, 2024). The sustainability of this mode is inherently constrained by its dependence on the planner's ongoing presence and influence. The induced behavior, more reflective of external regulation than intrinsic motivation, may falter once the hierarchical context changes, limiting its potential for fostering long-term habitual pro-environmental actions.

### Co-planned dyads: social interaction and identity fusion under power balance

In the collaborative planning combination, the power balance triggers deep social interaction, leading to the emergence of pro-environmental identity and pro-environmental behavior (Cao et al., 2025). Value conflicts force both parties to enter cognitive restructuring and reach consensus (Liberman and Chaiken, 1991). Both parties transform their differences into a common identity (such as “We are environmental partners”) through repeated negotiations (Ellemers et al., 2002). This jointly constructed “group commitment” has high stickiness (Ellemers et al., 2002; He et al., 2023). Essentially, collaborative planning is not a change in the decision-making method, but rather reshapes environmental actions from “You make me do it” to “We choose to do it together.” This is precisely the qualitative leap from external regulation to integrative regulation in the Self-Determination Theory (Howard et al., 2020; Núñez-Regueiro, 2024). In this process, co-planning addresses not only the “what to do” but, more profoundly, the “who we are” as a collective, resulting in behavior that is more resilient and self-sustaining as it becomes integrated into the individuals' self-concept, fulfilling core psychological needs for autonomy, relatedness, and competence.

### Implications for tourism: catalyzing environmental action and practical strategies

Although the partner effect of planners on followers is pronounced in contexts dominated by planners, such behavioral patterns still entail notable drawbacks. In hierarchical control scenarios characterized by planner-dominated dyads, the concentration of power enables the planner's environmental values to serve as a central behavioral influence, effectively to directly modify the actions of their partner. This top-down model, while empirically validating the penetration effect of power (Galinsky et al., 2003), results in the partner being ensnared in fragile compliance that ultimately undermined by extrinsic motivation (Kim, 2024).

In collaborative contexts, not only the partner effect significantly greater than that in the planner-dominated context, but there may also be deeper implications. In Symbiotic Emergence (Co-Planned Dyads), power parity fosters significant social negotiation, as conflicting values prompt cognitive restructuring (Liberman and Chaiken, 1991). Environmental values can influence their own and partners' pro-environmental identity, exerting a bidirectional reinforcing effect (Balunde et al., 2020; Gatersleben and Murtagh, 2012). This process culminates in the formation of co-created identities, such as “eco-partners,” and shared behavioral commitments (Ellemers et al., 2002). This may be capable of stimulating more sustained pro-environmental behavior, and future research could employ longitudinal data analysis of the actor-partner interdependence model (Balunde and Perlaviciute, 2023; Cao et al., 2025).

This study reveals that tourism transcends its conventional roles as an economic and cultural activity to serve as a catalyst for environmental protection, positioning travel as a viable pathway to “take green steps” toward sustainability (He et al., 2023). To enhance pro-environmental behavior, we propose two actionable strategies. First, in planner-dominated dyads (e.g., one partner solely planning the trip), elevating the planner's pro-environmental values through interventions like value-aligned video campaigns or targeted advertising that can trigger a cascading effect. This not only strengthens the planner's own pro-environmental identity but also fosters identity formation and behavioral adoption in their followers (via external regulation). Second, in co-planner dyads (e.g., joint itinerary design), enhancing social interaction (e.g., collaborative decision-making on routes or transportation) deepens mutual pro-environmental identity, thereby driving bidirectional pro-environmental behaviors. Complementing these, activity-based interventions (e.g., pro-environmental group activities like zero-waste hikes) further solidify shared identity through collective engagement, reinforcing both individual and paired pro-environmental actions. Therefore, tourism practitioners and policymakers should shift their perspective from viewing tourists as isolated individuals to actively shaping the quality of interactions between travel companions. By designing tourism products and services that necessitate collaboration (e.g., joint environmental tasks, negotiated green itineraries), the travel context can be transformed into a sustainability laboratory where pro-environmental behaviors are naturally adopted and reinforced through interaction.

### Methodological innovation, limitations, and future directions

From the actor-partner interdependence mode perspective, individual-level interventions (boosting planners' values) directly influence followers' identity and behavior, while collective-level emphasis on cooperation amplifies and sustains these effects over time. Methodologically, this work innovates by applying APIM to compare power-asymmetric dyads, revealing that co-planning generates stronger, more persistent pro-environmental behaviors than hierarchical planning (via path coefficient comparisons and group contrasts). Limitations include its retrospective design, which risks recall bias; future studies should adopt contextual experiments or real-time diary feedback to enhance ecological validity. Additionally, as a cross-sectional inquiry, longitudinal APIM designs are needed to verify whether cooperative models sustain their effects on pro-environmental behavior over time (e.g., tracking behavior at 6-month intervals to assess durability). These insights bridge theory and practice, underscoring travel's potential as a platform for scalable environmental action.

## Data Availability

The raw data supporting the conclusions of this article will be made available by the authors, without undue reservation.

## References

[B1] Ackerman R. Kenny D. (2016). APIMPowerR: An Interactive Tool for Actor-Partner Interdependence Model Power Analysis. Computer Software. Available online at: https://robert-a-ackerman.shinyapps.io/APIMPowerRdis/

[B2] Ajzen I. (1985). “From intentions to actions: a theory of planned behavior,” in Action Control: From Cognition to Behavior (Berlin; Heidelberg: Springer), 11–39. doi: 10.1007/978-3-642-69746-3_2

[B3] Anderson C. Brion S. (2014). Perspectives on power in organizations. Ann. Rev. Organ. Psychol. Organ. Behav. 1, 67–97. doi: 10.1146/annurev-orgpsych-031413-091259

[B4] Balunde A. Perlaviciute G. (2023). Are we on the same page? Exploring the relationships between environmental values, self-identity, personal norms and behavior in parent-adolescent dyads. J. Environ. Psychol. 92:102157. doi: 10.1016/j.jenvp.2023.102157

[B5] Balunde A. Perlaviciute G. Truskauskaite-Kuneviciene I. (2020). Sustainability in youth: environmental considerations in adolescence and their relationship to pro-environmental behavior. Front. Psychol. 11:582920. doi: 10.3389/fpsyg.2020.582920 33224073 PMC7667260

[B6] Bamberg S. Rees J. Seebauer S. (2015). Collective dimension: determinants of participation intention in community-based pro-environmental initiatives. J. Environ. Psychol. 43, 155–165. doi: 10.1016/j.jenvp.2015.06.006

[B7] Barbarossa C. De Pelsmacker P. Moons I. (2017). Personal values, green self-identity and electric car adoption. Ecol. Econ. 140, 190–200. doi: 10.1016/j.ecolecon.2017.05.015

[B8] Brauer M. Bourhis R. Y. (2006). Social power. Eur. J. Soc. Psychol. 36, 601–616. doi: 10.1002/ejsp.355

[B9] Cao X. Zhong F. Du M. (2025). Linking informal social interaction to residents' pro-environmental behaviors: evidence and implications for environmental policy making. J. Environ. Manag. 388:125923. doi: 10.1016/j.jenvman.2025.125923 40435803

[B10] Capiene A. Rutelione A. Krukowski K. (2022). Engaging in sustainable consumption: exploring the influence of environmental attitudes, values, personal norms, and perceived responsibility. Sustainability 14:10290. doi: 10.3390/su141610290

[B11] Carrico A. R. (2022). The promise of private-sphere pro-environmental behavior as climate action. Curr. Climate Change Rep. 8, 125–133. doi: 10.1007/s40641-022-00188-4

[B12] Chen F. F. (2007). Sensitivity of goodness of fit indexes to lack of measurement invariance. Struct. Eq. Model. Multidiscip. J. 14, 464–504. doi: 10.1080/10705510701301834

[B13] Chiu Y. T. H. Lee W. I. Chen T. H. (2013). Environmentally responsible behavior in ecotourism: antecedents and implications. Tourism Manag. 40, 321–329. doi: 10.1016/j.tourman.2013.06.013

[B14] Chwialkowska A. Bhatti W. A. Glowik M. (2020). The influence of cultural values on pro-environmental behavior. J. Clean. Prod. 268:122305. doi: 10.1016/j.jclepro.2020.122305

[B15] Cook W. L. Kenny D. A. (2005). The actor-partner interdependence model: a model of bidirectional effects in developmental studies. Int. J. Behav. Dev. 29, 101–109. doi: 10.1080/01650250444000405

[B16] Dewi W. Dian R S. (2018). Undergraduate students' pro-environmental behavior in daily practice. E3S Web Conf. 31:09025. doi: 10.1051/e3sconf/20183109025

[B17] Duncan O. D. (2014). Introduction to Structural Equation Models. Elsevier.

[B18] Dunlap R. E. Van Liere K. D. Mertig A. G. Jones R. E. (2000). Measuring endorsement of the new ecological paradigm: a revised NEP scale-statistical data included. J. Soc. Issues 56, 425–442. doi: 10.1111/0022-4537.00176

[B19] Ellemers N. Spears R. Doosje B. (2002). Self and social identity. Ann. Rev. Psychol. 53, 161–186. doi: 10.1146/annurev.psych.53.100901.135228 11752483

[B20] Galinsky A. D. Gruenfeld D. H. Magee J. C. (2003). From power to action. J. Personal. Soc. Psychol. 85:453. doi: 10.1037/0022-3514.85.3.453 14498782

[B21] Gao S. Li W. Ling S. Dou X. Liu X. (2019). An empirical study on the influence path of environmental risk perception on behavioral responses in China. Int. J. Environ. Res. Public Health 16:2856. doi: 10.3390/ijerph16162856 31405088 PMC6719179

[B22] Gatersleben B. Murtagh N. Abrahamse W. (2012). Values, identity and pro-environmental behaviour. Contemp. Soc. Sci. 9, 374–392. doi: 10.1080/21582041.2012.682086

[B23] He Y. Xu F. Wang L. Nguyen H. (2023). Modeling tourists' pro-environmental behavior a combination of the value-belief-norm theory and environmental identity theory. J. Environ. Plann. Manag. 67, 3694–3717. doi: 10.1080/09640568.2023.2232944

[B24] Howard J. L. Gagné M. Morin A. J. S. (2020). Putting the pieces together: reviewing the structural conceptualization of motivation within SDT. Motiv. Emot. 44, 846–861. doi: 10.1007/s11031-020-09838-2

[B25] Jaeger C. M. Schultz P. W. (2017). Coupling social norms and commitments: testing the underdetected nature of social influence. J. Environ. Psychol. 51, 199–208. doi: 10.1016/j.jenvp.2017.03.015

[B26] Karp D. G. (1996). Values and their effect on pro-environmental behavior. Environ. Behav. 28, 111–133. doi: 10.1177/0013916596281006

[B27] Kelley H. H. Holmes J. G. Kerr N. L. Reis H. T. Rusbult C. E. Van Lange P. A. (2003). An Atlas of Interpersonal Situations, Vol. 10. New York, NY: Cambridge University Press.

[B28] Khizar H. M. U. Younas A. Kumar S. Akbar A. Poulova P. (2023). The progression of sustainable development goals in tourism: a systematic literature review of past achievements and future promises. J. Innov. Knowl. 8:100442. doi: 10.1016/j.jik.2023.100442

[B29] Kim M. (2024). Signaling commitment via insincere conformity: a new take on the persistence of unpopular norms. Soc. Psychol. Q. 88, 111–134. doi: 10.1177/01902725241239953

[B30] Kühler M. (2021). “Love and conflicts between identity-forming values,” in International Handbook of Love (New York, NY: Springer International Publishing), 423–438. doi: 10.1007/978-3-030-45996-3_23

[B31] Ledermann T. Macho S. Kenny D. A. (2011). Assessing mediation in dyadic data using the Actor-partner interdependence model. Struct. Eq. Model. 18, 595–612. doi: 10.1080/10705511.2011.607099

[B32] Li C. Li L. Liu H. (2024). Decision as ritual: how power structure affects collective tourism destination decision-making. J. Hospit. Tour. Manag. 60, 373–383. doi: 10.1016/j.jhtm.2024.08.010

[B33] Liberman A. Chaiken S. (1991). Value conflict and thought-induced attitude change. J. Exp. Soc. Psychol. 27, 203–216. doi: 10.1016/0022-1031(91)90012-U

[B34] Magee J. C. (2020). Power and social distance. Curr. Opin. Psychol. 33, 33–37. doi: 10.1016/j.copsyc.06.005 31352249

[B35] Manner-Baldeon F. Lin G. Li M. (2023). Dyadic friendship travel: the role of personal and friendship characteristics on conflict management styles. J. Trav. Res. 63, 1219–1238. doi: 10.1177/00472875231184226

[B36] Merin Adelin E. Dimas Angga N. (2024). Pengaruh personal identity, social identity, kredibilitas influencer terhadap green purchasing behavior. Al-Kharaj: Jurnal Ekonomi, Keuangan andamp; Bisnis Syariah, 6:4945. doi: 10.47467/alkharaj.v6i9.4945

[B37] Núñez-Regueiro F. (2024). Cubic relations of autonomous and controlled motivation to achievement: a cross-national validation of self-determination theory using response surface analysis. Educ. Psychol. Rev. 36:2024. doi: 10.1007/s10648-024-09905-x

[B38] Ozyilmaz A. Koc S. (2022). Personal identity: how it moderates the relation between social identity and workplace performance. J. Manag. Organ. 30, 1845–1872. doi: 10.1017/jmo.2022.90

[B39] Ramos M. C. (2024). Values as master identities within identity structures. Soc. Psychol. Q. 88, 149–160. doi: 10.1177/01902725241289323

[B40] Sawitri D. R. Hadiyanto H. Hadi S. P. (2015). Pro-environmental behavior from a social cognitive theory perspective. Proced. Environ. Sci. 23, 27–33. doi: 10.1016/j.proenv.2015.01.005

[B41] Schwab S. D. Singh M. (2024). How power shapes behavior: evidence from physicians. Science 384, 802–808. doi: 10.1126/science.adl3855 38753782

[B42] Scoville J. N. (1995). Value theory and ecology in environmental ethics. Environ. Ethics 17, 115–133. doi: 10.5840/enviroethics199517225

[B43] Shekhovtsova V. A. (2020). (2020). Self-identification as a mechanism of personality identity formation. Humanit. Stud. Pedagog. Psychol. Philos. 3, 146–152. doi: 10.31548/hspedagog03.146

[B44] Smith C. J. Dupre K. E. McEvoy A. Kenny S. (2021). Community perceptions and pro-environmental behavior: the mediating roles of social norms and climate change risk. Can. J. Behav. Sci. 53, 200–210. doi: 10.1037/cbs0000229

[B45] Tumanggor R. O. Dariyo A. Subekti S. (2023). Ethical foundations of ecological behavior. Int. J. Appl. Soc. Sci. Humanit. 1, 58–66. doi: 10.24912/ijaassh.v1i1.25687

[B46] Turaga R. M. R. Howarth R. B. Borsuk M. E. (2010). Pro-environmental behavior. Ann. N. Y. Acad. Sci. 1185, 211–224. doi: 10.1111/j.1749-6632.2009.05163.x 20146771

[B47] Turner J. C. Reynolds K. J. (2003). “The social identity perspective in intergroup relations: theories, themes, and controversies,” in Blackwell Handbook of Social Psychology: Intergroup Processes 133–152. doi: 10.1002/9780470693421.ch7

[B48] Wallis H. Loy L. S. (2021). What drives pro-environmental activism of young people? A survey study on the Fridays For Future movement. J. Environ. Psychol. 74:101581. doi: 10.1016/j.jenvp.2021.101581

[B49] Xu L. Yang H. Ling M. (2022). Interpersonal contextual influences on the relationship between values and pro-environmental behaviors. Sustain. Product. Consumpt. 32, 532–540. doi: 10.1016/j.spc.2022.05.012

[B50] Yin Y. Savani K. Smith P. K. (2021). Power Increases perceptions of others' choices, leading people to blame others more. Soc. Psychol. Personal. Sci. 13, 170–177. doi: 10.1177/19485506211016140

[B51] Yuksel S. Y. (2021). The problem of subjectivity of values in the search for a universal environmental ethics. Herit. Sustain. Dev. 3, 53–57. doi: 10.37868/hsd.v3i1.54

[B52] Zeng Z. Zhong W. Naz S. (2023). Can environmental knowledge and risk perception make a difference? The role of environmental concern and pro-environmental behavior in fostering sustainable consumption behavior. Sustainability 15:4791. doi: 10.3390/su15064791

[B53] Zhang S. Yan K. (2025). Driving mechanism of pro-environmental donation intentions: an experimental study based on social norms and personal norms. Sustainability 18:268. doi: 10.3390/su18010268

[B54] Zhao A. L. Dermody J. Koenig-Lewis N. Hanmer-Lloyd S. (2023). Cultivating sustainable consumption: The role of harmonious cultural values and pro-environmental self-identity. J. Consum. Behav. 23, 1014–1031. doi: 10.1002/cb.2261

[B55] Zheng J. Yang M. Xu M. Zhao C. Shao C. (2019). An empirical study of the impact of social interaction on public pro-environmental behavior. Int. J. Environ. Res. Public Health 16:4405. doi: 10.3390/ijerph16224405 31717961 PMC6888221

[B56] Zhu Y. Wang Y. (2021). How does social interaction affect pro-environmental behaviors in China? The mediation role of conformity. Front. Environ. Sci. 9:690361. doi: 10.3389/fenvs.2021.690361

[B57] Zhu Y. Wang Y. Liu Z. (2021). How does social interaction affect pro-environmental behaviors in China? The mediation role of conformity. Front. Environ. Sci. 9:690361. doi: 10.3389/fenvs.2021.690361

