# Correction: Co-creating green steps: APIM evidence of mutual influence on pro-environmental behavior in travel pairs

**DOI:** 10.3389/fpsyg.2026.1822423

**Published:** 2026-03-18

**Authors:** Bopeng Yu, Jing Shi

**Affiliations:** School of Educational Science, Shenyang Normal University, Shenyang, China

**Keywords:** actor-partner interdependence, comparative research, environmental values, pro-environmental behavior, pro-environmental identity

In the published article, several references were incorrectly cited in the text. The affected citations are included below along with the correct citations:

The reference for [Ajzen, 1985] was erroneously written as [Ajzen, I. (1985)]. “From intentions to actions: a theory of planned behavior,” in *Action Control: From Cognition to Behavior*, eds. J. C. Turner and K. J. Reynolds (Berlin; Heidelberg: Springer), 11-39. doi: 10.1007/978-3-642-69746-3_2. It should be Ajzen, I. (1985). “From intentions to actions: a theory of planned behavior,” in *Action Control: From Cognition to Behavior* (Berlin; Heidelberg: Springer), 11–39. doi: 10.1007/978-3-642-69746-3_2.

The reference for Anderson, 2014 was erroneously written as Anderson, C. (2014). Perspectives on power in organizations. *Ann. Rev. Organ. Psychol. Organ. Behav*. 1, 67–97. doi: 10.1146/annurev-orgpsych-031413-091259. It should be Anderson, C., and Brion, S. (2014). Perspectives on power in organizations. *Ann. Rev. Organ. Psychol. Organ. Behav*. 1, 67–97. doi: 10.1146/annurev-orgpsych-031413-091259.

The reference for Balunde, 2023 was erroneously written as Balunde, A. (2023). Are we on the same page? Exploring the relationships between environmental values, self-identity, personal norms and behavior in parent-adolescent dyads. *J. Environ. Psychol*. 92:102157. doi: 10.1016/j.jenvp.2023.102157. It should be Balunde, A., and Perlaviciute, G. (2023). Are we on the same page? Exploring the relationships between environmental values, self-identity, personal norms and behavior in parent-adolescent dyads. *J. Environ. Psychol*. 92:102157. doi: 10.1016/j.jenvp.2023.102157.

The reference for Balunde and Perlaviciute, 2020 was erroneously written as Balunde, A., and Perlaviciute, G. (2020). Sustainability in youth: environmental considerations in adolescence and their relationship to pro-environmental behavior. *Front. Psychol*. 11:582920. doi: 10.3389/fpsyg.2020.582920. It should be Balunde, A., Perlaviciute, G., and Truskauskaite-Kuneviciene, I. (2020). Sustainability in youth: environmental considerations in adolescence and their relationship to pro-environmental behavior. *Front. Psychol*. 11:582920. doi: 10.3389/fpsyg.2020.582920.

The reference for Bamberg and Rees, 2015 was erroneously written as Bamberg, S., and Rees, J. (2015). Collective dimension: determinants of participation intention in community-based pro-environmental initiatives. *J. Environ. Psychol*. 43, 155–165. doi: 10.1016/j.jenvp.2015.06.006. It should be Bamberg, S., Rees, J., and Seebauer, S. (2015). Collective dimension: determinants of participation intention in community-based pro-environmental initiatives. *J. Environ. Psychol*. 43, 155–165. doi: 10.1016/j.jenvp.2015.06.006.

The reference for Barbarossa and Pelsmacker, 2017 was erroneously written as Barbarossa, C., and Pelsmacker, D. (2017). Personal values, green self-identity and electric car adoption. *Ecol. Econ*. 140, 190–200. doi: 10.1016/j.ecolecon.2017.05.015. It should be Barbarossa, C., De Pelsmacker, P., and Moons, I. (2017). Personal values, green self-identity and electric car adoption. *Ecol. Econ*. 140, 190–200. doi: 10.1016/j.ecolecon.2017.05.015.

The reference for Brauer, 2006 was erroneously written as Brauer, M. (2006). Social power. *Eur. J. Soc. Psychol*. 36, 601–616. doi: 10.1002/ejsp.355. It should be Brauer, M., and Bourhis, R. Y. (2006). Social power. *Eur. J. Soc. Psychol*. 36, 601–616. doi: 10.1002/ejsp.355.

The reference for Cao and Zhong, 2025 was erroneously written as Cao, X., and Zhong, F. (2025). Linking informal social interaction to residents' pro-environmental behaviors: evidence and implications for environmental policy making. *J. Environ. Manag*. 388:125923. doi: 10.1016/j.jenvman.2025.125923. It should be Cao, X., Zhong, F., and Du, M. (2025). Linking informal social interaction to residents' pro-environmental behaviors: evidence and implications for environmental policy making. *J. Environ. Manag*. 388:125923. doi: 10.1016/j.jenvman.2025.125923.

The reference for Capiene and Rutelione, 2022 was erroneously written as Capiene, A., and Rutelione, A. (2022). Engaging in sustainable consumption: exploring the influence of environmental attitudes, values, personal norms, and perceived responsibility. *Sustainability* 14:10290. doi: 10.3390/su141610290. It should be Capiene, A., Rutelione, A., and Krukowski, K. (2022). Engaging in sustainable consumption: exploring the influence of environmental attitudes, values, personal norms, and perceived responsibility. *Sustainability* 14:10290. doi: 10.3390/su141610290.

The reference for Chiu and Lee, 2013 was erroneously written as Chiu, Y. T. H., and Lee, W. I. (2013). Environmentally responsible behavior in ecotourism: antecedents and implications. *Tourism Manag*. 40, 321–329. doi: 10.1016/j.tourman.2013.06.013. It should be Chiu, Y. T. H., Lee, W. I., and Chen, T. H. (2013). Environmentally responsible behavior in ecotourism: antecedents and implications. *Tourism Manag*. 40, 321–329. doi: 10.1016/j.tourman.2013.06.013.

The reference for Chwialkowska and Bhatti, 2020 was erroneously written as Chwialkowska, A., and Bhatti, W. A. (2020). The influence of cultural values on pro-environmental behavior. *J. Clean. Prod*. 268:122305. doi: 10.1016/j.jclepro.2020.122305. It should be Chwialkowska, A., Bhatti, W. A., and Glowik, M. (2020). The influence of cultural values on pro-environmental behavior. *J. Clean. Prod*. 268:122305. doi: 10.1016/j.jclepro.2020.122305.

The reference for Cook, 2005 was erroneously written as Cook, W. L. (2005). The actor-partner interdependence model: a model of bidirectional effects in developmental studies. *Int. J. Behav. Dev*. 29, 101–109. doi: 10.1080/01650250444000405. It should be Cook, W. L., and Kenny, D. A. (2005). The actor-partner interdependence model: a model of bidirectional effects in developmental studies. *Int. J. Behav. Dev*. 29, 101–109. doi: 10.1080/01650250444000405.

The reference for Dewi, 2018 was erroneously written as Dewi, W. S. (2018). Undergraduate students' pro-environmental behavior in daily practice. *E3S Web of Conf*. 31:09025. doi: 10.1051/e3sconf/20183109025. It should be Dewi, W., and Dian R, S. (2018). Undergraduate students' pro-environmental behavior in daily practice. *E3S Web Conf*. 31:09025. doi: 10.1051/e3sconf/20183109025.

The reference for Dunlap et al., 2000 was erroneously written as Dunlap, R. E., Van Liere, K. D., and Mertig, A. G. (2000). Measuring endorsement of the new ecological paradigm: a revised NEP scale-statistical data included. *J. Soc. Issues* 56, 425–442. doi: 10.1111/0022-4537.00176. It should be Dunlap, R. E., Van Liere, K. D., Mertig, A. G., and Jones, R. E. (2000). Measuring endorsement of the new ecological paradigm: a revised NEP scale-statistical data included. *J. Soc. Issues* 56, 425–442. doi: 10.1111/0022-4537.00176.

The reference for Ellemers and Spears, 2002 was erroneously written as Ellemers, N., and Spears, R. (2002). Self and social identity. *Ann. Rev. Psychol*. 53, 161–186. doi: 10.1146/annurev.psych.53.100901.135228. It should be Ellemers, N., Spears, R., and Doosje, B. (2002). Self and social identity. *Ann. Rev. Psychol*. 53, 161–186. doi: 10.1146/annurev.psych.53.100901.135228.

The reference for Galinsky and Gruenfeld, 2003 was erroneously written as Galinsky, A. D., and Gruenfeld, D. H. (2003). From power to action. *J. Personal. Soc. Psychol*. 85:453. doi: 10.1037/0022-3514.85.3.453. It should be Galinsky, A. D., Gruenfeld, D. H., and Magee, J. C. (2003). From power to action. *J. Personal. Soc. Psychol*. 85:453. doi: 10.1037/0022-3514.85.3.453.

The reference for Gao et al., 2019 was erroneously written as Gao, S., Li, W., Ling, S., and Dou, X. (2019). An empirical study on the influence path of environmental risk perception on behavioral responses in China. *Int. J. Environ. Res. Public Health* 16:2856. doi: 10.3390/ijerph16162856. It should be Gao, S., Li, W., Ling, S., Dou, X., and Liu, X. (2019). An empirical study on the influence path of environmental risk perception on behavioral responses in China. *Int. J. Environ. Res. Public Health* 16:2856. doi: 10.3390/ijerph16162856.

The reference for Gatersleben and Murtagh, 2012 was erroneously written as Gatersleben, B., and Murtagh, N. (2012). Values, identity and pro-environmental behaviour. *Contemp. Soc. Sci*. 9, 374–392. doi: 10.1080/21582041.2012.682086. It should be Gatersleben, B., Murtagh, N., and Abrahamse, W. (2012). Values, identity and pro-environmental behaviour. *Contemp. Soc. Sci*. 9, 374–392. doi: 10.1080/21582041.2012.682086.

The reference for He et al., 2023 was erroneously written as He, Y., Xu, F., and Wang, L. (2023). Modeling tourists' pro-environmental behavior a combination of the value-belief-norm theory and environmental identity theory. *J. Environ. Plann. Manag*. 67, 3694–3717. doi: 10.1080/09640568.2023.2232944. It should be He, Y., Xu, F., Wang, L., and Nguyen, H. (2023). Modeling tourists' pro-environmental behavior a combination of the value-belief-norm theory and environmental identity theory. *J. Environ. Plann. Manag*. 67, 3694–3717. doi: 10.1080/09640568.2023.2232944.

The reference for Howard and Gagne, 2020 was erroneously written as Howard, J. L., and Gagne, M. (2020). Putting the pieces together: reviewing the structural conceptualization of motivation within SDT. *Motiv. Emot*. 44, 846–861. doi: 10.1007/s11031-020-09838-2. It should be Howard, J. L., Gagné, M., and Morin, A. J. S. (2020). Putting the pieces together: reviewing the structural conceptualization of motivation within SDT. *Motiv. Emot*. 44, 846–861. doi: 10.1007/s11031-020-09838-2.

The reference for Jaeger, 2017 was erroneously written as Jaeger, C. M. (2017). Coupling social norms and commitments: testing the underdetected nature of social influence. *J. Environ. Psychol*. 51, 199–208. doi: 10.1016/j.jenvp.2017.03.015. It should be Jaeger, C. M., and Schultz, P. W. (2017). Coupling social norms and commitments: testing the underdetected nature of social influence. *J. Environ. Psychol*. 51, 199–208. doi: 10.1016/j.jenvp.2017.03.015.

The reference for Kelley et al., 2003 was erroneously written as Kelley, H. H., Holmes, J. G., Kerr, N. L., Reis, H. T., and Rusbult, C. E. (2003). *An Atlas of Interpersonal Situations*, Vol. 10. New York, NY: Cambridge University Press. It should be Kelley, H. H., Holmes, J. G., Kerr, N. L., Reis, H. T., Rusbult, C. E., and Van Lange, P. A. (2003). *An Atlas of Interpersonal Situations*, Vol. 10. New York, NY: Cambridge University Press.

The reference for Khizar et al., 2023 was erroneously written as Khizar, H. M. U., Younas, A., Kumar, S., and Akbar, A. (2023). The progression of sustainable development goals in tourism: a systematic literature review of past achievements and future promises. *J. Innov. Knowl*. 8:100442. doi: 10.1016/j.jik.2023.100442. It should be Khizar, H. M. U., Younas, A., Kumar, S., Akbar, A., and Poulova, P. (2023). The progression of sustainable development goals in tourism: a systematic literature review of past achievements and future promises. *J. Innov. Knowl*. 8:100442. doi: 10.1016/j.jik.2023.100442.

The reference for Ledermann and Macho, 2011 was erroneously written as Ledermann, T., and Macho, S. (2011). Assessing mediation in dyadic data using the Actor-partner interdependence model. *Struct. Eq. Model*. 18, 595–612. doi: 10.1080/10705511.2011.607099. It should be [Ledermann, T., Macho, S., and Kenny, D. A. (2011)]. Assessing mediation in dyadic data using the Actor-partner interdependence model. *Struct. Eq. Model*. 18, 595–612. doi: 10.1080/10705511.2011.607099.

The reference for [Li and Li, 2024] was erroneously written as [Li, C., and Li, L. (2024)]. Decision as ritual: how power structure affects collective tourism destination decision-making. *J. Hospit. Tour. Manag*. 60, 373–383. doi: 10.1016/j.jhtm.2024.08.010. It should be [Li, C., Li, L., and Liu, H. (2024)]. Decision as ritual: how power structure affects collective tourism destination decision-making. *J. Hospit. Tour. Manag*. 60, 373–383. doi: 10.1016/j.jhtm.2024.08.010.

The reference for [Liberman, 1991] was erroneously written as [Liberman, A. (1991)]. Value conflict and thought-induced attitude change. *J. Exp. Soc. Psychol*. 27, 203–216. doi: 10.1016/0022-1031(91)90012-U. It should be [Liberman, A., and Chaiken, S. (1991)]. Value conflict and thought-induced attitude change. *J. Exp. Soc. Psychol*. 27, 203–216. doi: 10.1016/0022-1031(91)90012-U.

The reference for [Manner-Baldeon and Lin, 2023] was erroneously written as [Manner-Baldeon, F., and Lin, G. (2023)]. Dyadic friendship travel: the role of personal and friendship characteristics on conflict management styles. *J. Trav. Res*. 63, 1219–1238. doi: 10.1177/00472875231184226. It should be [Manner-Baldeon, F., Lin, G., and Li, M. (2023)]. Dyadic friendship travel: the role of personal and friendship characteristics on conflict management styles. *J. Trav. Res*. 63, 1219–1238. doi: 10.1177/00472875231184226.

The reference for [Ozyilmaz 2022] was erroneously written as [Ozyilmaz, A. (2022)]. Personal identity: how it moderates the relation between social identity and workplace performance. *J. Manag. Organ*. 30, 1845–1872. doi: 10.1017/jmo.2022.90. It should be [Ozyilmaz, A., and Koc, S. (2022)]. Personal identity: how it moderates the relation between social identity and workplace performance. *J. Manag. Organ*. 30, 1845–1872. doi: 10.1017/jmo.2022.90.

The reference for [Sawitri and Hadiyanto, 2015] was erroneously written as [Sawitri, D. R., and Hadiyanto, H. (2015)]. Pro-environmental behavior from a social cognitive theory perspective. *Proced. Environ. Sci*. 23, 27–33. doi: 10.1016/j.proenv.2015.01.005. It should be [Sawitri, D. R., Hadiyanto, H., and Hadi, S. P. (2015)]. Pro-environmental behavior from a social cognitive theory perspective. *Proced. Environ. Sci*. 23, 27–33. doi: 10.1016/j.proenv.2015.01.005.

The reference for [Schwab, 2024] was erroneously written as [Schwab, S. D. (2024)]. How power shapes behavior: evidence from physicians. *Science* 384, 802–808. doi: 10.1126/science.adl3855. It should be [Schwab, S. D., and Singh, M. (2024)]. How power shapes behavior: evidence from physicians. *Science* 384, 802–808. doi: 10.1126/science.adl3855.

The reference for [Smith et al., 2021] was erroneously written as [Smith, C. J., Dupré, K. E., and McEvoy, A. (2021)]. Community perceptions and pro-environmental behavior: the mediating roles of social norms and climate change risk. *Can. J. Behav. Sci*. 53, 200–210. doi: 10.1037/cbs0000229. It should be [Smith, C. J., Dupre, K. E., McEvoy, A., and Kenny, S. (2021). Community perceptions and pro-environmental behavior: the mediating roles of social norms and climate change risk. *Can. J. Behav. Sci*. 53, 200–210. doi: 10.1037/cbs0000229.

The reference for Tumanggo and Dariyo, 2023 was erroneously written as Tumanggor, R. O., and Dariyo, A. (2023). Ethical foundations of ecological behavior. *Int. J. Appl. Soc. Sci. Humanit*. 1, 58–66. doi: 10.24912/ijaassh.v1i1.25687. It should be Tumanggor, R. O., Dariyo, A., and Subekti, S. (2023). Ethical foundations of ecological behavior. *Int. J. Appl. Soc. Sci. Humanit*. 1, 58–66. doi: 10.24912/ijaassh.v1i1.25687.

The reference for Turaga and Howarth, 2010 was erroneously written as Turaga, R. M. R., and Howarth, R. B. (2010). Pro-environmental behavior. *Ann. New York Acad. Sci*. 1185, 211–224. doi: 10.1111/j.1749-6632.2009.05163.x. It should be Turaga, R. M. R., Howarth, R. B., and Borsuk, M. E. (2010). Pro-environmental behavior. *Ann. N. Y. Acad. Sci*. 1185, 211–224. doi: 10.1111/j.1749-6632.2009.05163.x.

The reference for Wallis, 2021 was erroneously written as Wallis, H. (2021). What drives pro-environmental activism of young people? A survey study on the Fridays For Future movement. *J. Environ. Psychol*. 74:101581. doi: 10.1016/j.jenvp.2021.101581. It should be [Wallis, H., and Loy, L. S. (2021). What drives pro-environmental activism of young people? A survey study on the Fridays For Future movement. *J. Environ. Psychol*. 74:101581. doi: 10.1016/j.jenvp.2021.101581.

The reference for Xu and Yang, 2022 was erroneously written as Xu, L., and Yang, H. (2022). Interpersonal contextual influences on the relationship between values and pro-environmental behaviors. *Sustain. Product. Consumpt*. 32, 532–540. doi: 10.1016/j.spc.2022.05.012. It should be [Xu, L., Yang, H., and Ling, M. (2022). Interpersonal contextual influences on the relationship between values and pro-environmental behaviors. *Sustain. Product. Consumpt*. 32, 532–540. doi: 10.1016/j.spc.2022.05.012.

The reference for Yin and Savani, 2021 was erroneously written as Yin, Y., and Savani, K. (2021). Power Increases perceptions of others' choices, leading people to blame others more. *Soc. Psychol. Personal. Sci*. 13, 170–177. doi: 10.1177/19485506211016140. It should be Yin, Y., Savani, K., and Smith, P. K. (2021). Power Increases perceptions of others' choices, leading people to blame others more. *Soc. Psychol. Personal. Sci*. 13, 170–177. doi: 10.1177/19485506211016140.

The reference for Zeng and Zhong, 2023 was erroneously written as Zeng, Z., and Zhong, W. (2023). Can environmental knowledge and risk perception make a difference? The role of environmental concern and pro-environmental behavior in fostering sustainable consumption behavior. *Sustainability* 15:4791. doi: 10.3390/su15064791. It should be Zeng, Z., Zhong, W., and Naz, S. (2023). Can environmental knowledge and risk perception make a difference? The role of environmental concern and pro-environmental behavior in fostering sustainable consumption behavior. *Sustainability* 15:4791. doi: 10.3390/su15064791.

The reference for Zheng et al., 2019 was erroneously written as Zheng, J., Yang, M., Xu, M., and Zhao, C. (2019). An empirical study of the impact of social interaction on public pro-environmental behavior. *Int. J. Environ. Res. Public Health* 16:4405. doi: 10.3390/ijerph16224405. It should be Zheng, J., Yang, M., Xu, M., Zhao, C., and Shao, C. (2019). An empirical study of the impact of social interaction on public pro-environmental behavior. *Int. J. Environ. Res. Public Health* 16:4405. doi: 10.3390/ijerph16224405.

The reference for Zhu and Wang, 2021 was erroneously written as Zhu, Y., and Wang, Y. (2021). How does social interaction affect pro-environmental behaviors in China? The mediation role of conformity. *Front. Environ. Sci*. 9:690361. doi: 10.3389/fenvs.2021.690361. It should be Zhu, Y., Wang, Y., and Liu, Z. (2021). How does social interaction affect pro-environmental behaviors in China? The mediation role of conformity. *Front. Environ. Sci*. 9:690361. doi: 10.3389/fenvs.2021.690361.

The original version of this article has been updated.

