# Supplementary material for: Co-creating green steps: APIM evidence of mutual influence on pro-environmental behavior in travel pairs
Source: Front Psychol. 2026 Mar 2;17:1730412. doi: 10.3389/fpsyg.2026.1730412 (PMC12989399; doi:10.3389/fpsyg.2026.1730412)
Supplement: Supplementary file 1 [file Table_1.docx]

Supplementary Material

**Appendix A. Questionnaire**

**Environmental values**

1. We are approaching the limit of the number of people the earth can support.

2. Humans have the right to modify the natural environment to suit their needs.

3. When humans interfere with nature it often produces disastrous consequences.

4. Human ingenuity will insure that we do NOT make the earth unlivable.

5. Humans are severely abusing the environment.

6. The earth has plenty of natural resources if we just learn how to develop them.

7. Plants and animals have as much right as humans to exist.

8. The balance of nature is strong enough to cope with the impacts of modern industrial nations.

9. Despite our special abilities humans are still subject to the laws of nature.

10. The so-called “ecological crisis” facing humankind has been greatly exaggerated.

11. The earth is like a spaceship with very limited room and resources.

12. Humans were meant to rule over the rest of nature.

13. The balance of nature is very delicate and easily upset.

14. Humans will eventually learn enough about how nature works to be able to control it.

15. If things continue on their present course, we will soon experience a major ecological catastrophe.

**Pro-environmental identity**

1. I identify with others who engage in environmental protection.

2. I feel a strong connection to others who engage in environmental protection.

3. I feel proud to be active to protect the environment together with others.

**Pro-environmental behavior**

1. I comply with the site access regulations and management rules of the tourism destination.

2. I assist in maintaining the environmental quality of the tourist site.

3. I report any environmental pollution or destructive activities to the management authorities.

4. I spend money on goods and services within the local community.

5. I help other tourists to learn about the local environment and related knowledge.

6. I sort my trash at the travel site.

7. I make a conscious effort to minimize disruption to the natural ecology during my travels.
